# Proof of Concept for Cell Culture-Based Coffee

**DOI:** 10.1021/acs.jafc.3c04503

**Published:** 2023-11-16

**Authors:** Heikki Aisala, Elviira Kärkkäinen, Iina Jokinen, Tuulikki Seppänen-Laakso, Heiko Rischer

**Affiliations:** VTT Technical Research Centre of Finland Ltd, P.O. Box 1000, Espoo FI-02044, Finland

**Keywords:** *Coffea*, alternative coffee, beverage, biotechnology, cellular agriculture, bioreactor, plant cell

## Abstract

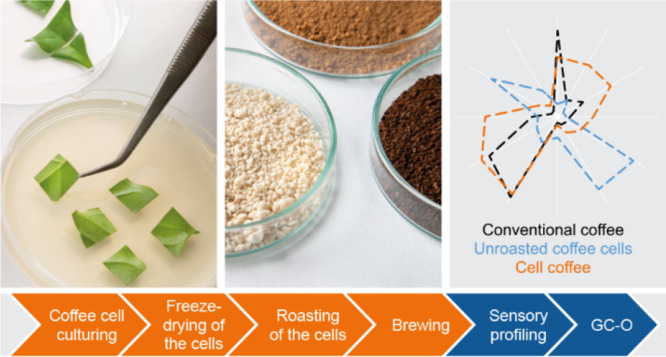

The global coffee production is facing serious challenges including land use, climate change, and sustainability while demand is rising. Cellular agriculture is a promising alternative to produce plant-based commodities such as coffee, which are conventionally produced by farming. In this study, the complex process of drying and roasting was adapted for bioreactor-grown coffee cells to generate a coffee-like aroma and flavor. The brews resulting from different roasting regimes were characterized with chemical and sensory evaluation-based approaches and compared to conventional coffee. Roasting clearly influenced the aroma profile. In contrast to conventional coffee, the dominant odor and flavor attributes were burned sugar-like and smoky but less roasted. The intensities of bitterness and sourness were similar to those of conventional coffee. The present results demonstrate a proof of concept for a cellular agriculture approach as an alternative coffee production platform and guide future optimization work.

## 1 Introduction

Coffee is one of the most consumed beverages in the world. Its raw material, green coffee beans, constitutes one of the most widely traded agricultural commodities. Annually, over 9.5 billion kg are produced with a corresponding total trade value of over $30 billion.^1^ Harvest yields are strongly affected by regional weather events with floods and droughts in major producer countries frequently impacting the highly volatile market.^2^ Compared to other agricultural products, coffee has a high carbon footprint accounting globally to 33–126 billion kg CO_2_ per annum, which is in the range of the annual emissions of countries like Denmark and the Philippines.^3^ Although 124 coffee species are recognized by botanists, only Arabica (*Coffea arabica*) and Robusta (*Coffea canephora*) account for the global trade with 60% and 40%, respectively.^4^ Coffee grows in the so-called “coffee belt” around the equator and optimally at specific altitudes from 1300 to 1600 m. Outside this range, pests, especially fungal infections such as coffee leaf rust, put the cultivation under pressure^5^ and necessitate heavy pesticide use. Future plant breeding is severely hampered by the fact that most wild coffee species, which are only known from Africa and South Asia, are at risk of extinction.^4^ With demand for coffee expected to triple by 2050^6^ and predictions that global production will decrease by half in the next 30 years due to the impacts of global climate change,^7^ it is apparent that coffee cultivation faces an uncertain future.

Biotechnology bears potential to tackle both challenges simultaneously–reducing environmental impact while sustaining production. Cellular agriculture, that is, the contained cultivation of cells in bioreactors to produce agricultural commodities rather than production by farmed animals or crops, has already made an impact in several sectors.^8^ The favorable environmental footprint of cultured plant cells instead of plants has already been evaluated for tobacco and cloudberry cell cultures^9^ but not yet for coffee cells. Most interestingly, the idea to substitute green coffee beans with cultured coffee cells had already been pioneered by Townsley^10^ in 1974. In this visionary paper, the author described the small-scale generation of coffee cells in the laboratory and claimed that the roasted material produces aroma and taste characteristics identical to conventional coffee. However, the flavor of coffee is very complex, with various volatile and nonvolatile compounds responsible for the resulting sensory properties.^11−13^ The chemical composition of the brew is influenced by numerous variables. These include not only the provenance, cultivar, and maturity level of the green bean^11,14^ but also various parameters during roasting and brewing such as temperature profile, particle size, grind to water ratio, water temperature, and the brewing method.^11,13,15−17^

Coffee aroma and flavor constitute the most important parameters for the consumer. Despite the encouraging message of the publication by Townsley, there is a lack of details and data related to the aroma and flavor of cell-derived coffee. For this reason, we revisit the concept and provide detailed results based on sensory evaluation and analytical investigation of roasted coffee cells.

## 2 Methods

### 2.1 Coffee Cell Culture

Commercial *Coffea arabica* seedlings (Plantagen Finland Oy, Vantaa, Finland) served as a source for leaf explants. Young but fully developed leaves were cut off and kept at room temperature to allow for stomata closure. The leaves were dipped in 70% ethanol for 2 min and then submerged in a 2.4% NaOCl solution with three drops of Tween 20 for 10 min followed by rinsing four times in sterile water. Then, the leaves were cut in squares of approximately 0.5 cm^2^ and placed with the adaxial side on callus induction medium (Supporting Information Table S1).^18^ The plates were incubated at 24 °C in darkness. After 2 to 4 weeks, callus had formed and was cut off from the explants to be subcultured on callus establishment medium (Supporting Information Table S1). For maintenance, the calli were subcultured monthly on callus maintenance medium (Supporting Information Table S1). Cell suspensions were established in the same medium without a gelling agent in 250 mL Erlenmeyer flasks containing 70 mL of medium on an orbital shaker at 110 rpm and 24 °C in darkness. The subculturing rhythm of the coffee cell suspension was 10 days.

For biomass production, coffee cells were cultivated in a wave bioreactor (Biostat RM, Sartorius, Germany) using 20 and 50 L wave bags (CultiBag RM, 20 L, 50 L basic, Sartorius, Germany) with final working volumes of 10 and 25 L, respectively. Cultivation parameters were adjusted as follows: temperature 24 °C, angle 10°, rocking level 26 for 20 L wave and 24 for 50 L wave, aeration 300 mL/min, in darkness. Inoculums for wave bag cultivation were prepared in shake flasks.

Plant cell biomass was harvested by filtering with Miracloth (Calbiochem, San Diego, USA) in a Buchner funnel and subsequently washed with sterile water. Cells were frozen and lyophilized (Epsilon 2–25 freeze-dryer, Martin Christ Gefriertrocknungsanlagen GmbH, Osterode am Harz, Germany). Freeze-dried cells were stored in airtight containers in a freezer at −20 °C until roasting.

### 2.2 Roasting of Coffee Cells

Roasting of the coffee cell powder was done in a fan-assisted oven (Electrolux FCE061, Electrolux Professional SpA, Viale Treviso, Italy) on a wire rack at 225 °C. Twenty-five grams of freeze-dried cells was weighed and moved to a bag made of greaseproof paper (Serla Leivinpaperi, Metsä Tissue Oyj, Mänttä, Finland). Three different roasting conditions were prepared, all including two parallel bags of cell material. For roasting condition (1), cells were first kept in the oven for 6 min and were mixed every 30 s to ensure homogeneous roasting. Cells were allowed to completely cool down, and roasting was then continued for another 14 min with mixing at 1 min intervals. For condition (2), cells were roasted in total for 12 min with mixing every 30 s. For condition (3), roasting took 15 min in total with mixing every 30 s for the first 2 min and then at 1 min intervals. Parallel roasting bags of the same batch were pooled together, and the powder was thoroughly mixed and transferred to foil bags. The bags were sealed and stored at room temperature.

### 2.3 Reference Coffee Samples

Three conventional, commercially available coffee products or coffee substitutes were purchased for the study. Commercial, dark roasted (roast level 4 in the Finnish roast grading system, ranging from 1–5) Arabica coffee beans and grinds (Pelican Rouge Rich Blend, Pelican Rouge Coffee Solutions Oy, Vantaa, Finland) were used in the color, toxicity, and sensory analyses. Commercial, light roasted (roast level 1), and ground Arabica coffee beans (Juhla Mokka, Oy Paulig Finland AB, Helsinki, Finland) were used in the color and sensory analyses. Instant chicory coffee (Chikko not coffee, Ghee Easy B.V., Amsterdam, The Netherlands) was used in the sensory analysis.

Additionally, green unroasted coffee beans were studied to demonstrate that the roasting process causes similar changes in preground coffee material as in the novel cell coffee material. Green beans of the cultivar Colombia Narino Excelso (Coffee Greens ApS, Hellerup, Denmark) were used as samples for color, volatile compound, and hydroxycinnamic acid analyses. For roasting, the beans were ground in a Retsch mixer mill, sieved through a 1.0 mm sieve, and roasted at 225 °C either for 6, 8, or 10 min with 1 min mixing intervals to create samples with similar color values (section 2.8) as the roasted coffee cells.

### 2.4 Microbiological Assays

To ensure the safety of brewed samples evaluated by the sensory panel, microbiological analyses were carried out. Samples were taken from the suspension cultures to confirm sterile culture conditions and from brewed and filtered samples tested in sensory evaluations. All samples were plated on PCA (plate count agar) and PDA (potato dextrose agar) plates. The plates were incubated for 3 days at 28 °C and for 5 days at 25 °C. To accept the plant cell culture samples for sensory evaluation, the limit of microbial count was set to less than 10 or less than 1 CFU/mL.

### 2.5 Acute Toxicity Analysis

Acute toxicity of unroasted and roasted coffee cells was studied using freshwater crustaceans *Daphnia magna* according to the DAPHTOXKIT F Magna (ISO 6341, Standard Operational Procedure (MicroBioTests Inc., Belgium)). Commercial, dark roasted, and ground Arabica coffee beans (Pelican Rouge Rich Blend) were included in the analysis for comparison.

In the case of coffee cells, 1.5 g of freeze-dried and roasted material was extracted with 30 mL of RO-water. The hydrothermal extraction proceeded for 30 min at 80 °C in a water bath. After the supernatant was removed, the residue was re-extracted with the same procedure. Each fraction was then centrifuged for 10 min at room temperature and 3220 x *g* (Eppendorf Centrifuge 5810 R, Eppendorf AG, Hamburg, Germany). The supernatants were pooled, lyophilized, and stored at −20 °C. In the case of the commercial coffee sample, the same procedure was used, but the ground coffee was first freeze-dried to extract comparable amounts.

Test solutions were prepared by dissolving the extract samples in Standard Freshwater (SF; provided with the test kit) at a concentration of 2.0 mg/mL, and the pH was measured and recorded. Then, the solutions were stepwise diluted to 1.0, 0.5, and 0.25 mg/mL concentrations. SF was used as a positive control and a K_2_Cr_2_O_7_ solution as a negative control.

Tests were performed according to the instructions of the test kit. Shortly, dormant ephippia of *Daphnia magna* were released in SF water and allowed to develop for 3 days under strong illumination at 20 °C. Hatched neonates were prefed with *Spirulina* powder 2 h before starting the actual experiment. In the test, five *Daphnia* neonates per well were used for each sample in a multiwell plate. Samples with *Daphnia* neonates were incubated at 20 °C in darkness. The number of viable and dead neonates was recorded after 24 and 48 h. The toxicity of the test solutions was calculated from the ratio of dead or immobilized crustaceans against the total count and expressed as the EC50 values (effective concentration).

### 2.6 Caffeine and Hydroxycinnamic Acid Analyses

For caffeine analysis, 20 mg aliquots (range 20.11–21.27 mg) of freeze-dried coffee cells or ground coffee beans were mixed with water (2 mL with beans, 3 mL with cells) and heated at 60 °C for 30 min. The samples were filtrated with PALL Acrodisc 25 mm syringe filters with 0.2 μm WWPTFE membrane (PALL, New York, USA), and the filtrates were diluted and analyzed for caffeine on a UPLC-Xevo TQ-S-MS (Waters, Milford, USA) by using the MRM technique in ESI positive ion mode (transition *m*/*z* 195.2 → 138.0). The UPLC column used was a Gemini C18 (50 × 2.0 mm, 3 μm), and the flow rate was 0.5 mL/min. The gradient was as follows: from initial to 0.30 min 98% solvent A (0.1% formic acid in water) and 2% of B (0.1% formic acid in acetonitrile), at 5.50 min 90% A and 10% B, from 7.70 to 8.70 min 50% A and 50% B, from 8.71 to 9.70 min 10% A and 90% B, and back to the initial conditions. The total run time was 12 min.

The MS conditions were as follows: capillary 3.1 kV, cone 20 V, source temperature 150 °C, desolvation gas flow 1000 L/h and desolvation temperature 500 °C, cone gas 150 L/h, and MSMS collision energy 20 V.

Quantification was based on a calibration curve determined for a caffeine reference compound (Merck 102584, Darmstadt, Germany) within a concentration range of 0.1–6 μg/mL.

The levels of major hydroxycinnamic acids were determined from 50 mg (DW) aliquots of coffee beans and cell samples. After alkaline hydrolysis (2 M NaOH), the samples were acidified and extracted with ethyl acetate. The extracts were evaporated, dissolved in 50% MeOH, and subjected to UPLC-DAD-QTof-MS analysis. The quinic acid esters of caffeic-, ferulic-, and *p*-coumaric acids were determined in MeOH extracts obtained from 50 mg (DW) samples. Quantification was based on external calibration with ferulic acid (Aldrich Chemistry/Merck KGaA, Darmstadt, Germany) and chlorogenic acid (Extrasynthese, Genay, France). Literature data, reference substances, and mass fragmentation data were used for identification (Supporting Information Table S2).

### 2.7 Sample Preparation and Serving for Sensory Analysis

In a beaker, 6 g of coffee cells or ground coffee was covered with 100 mL of 98 °C tap water. The ratio of 5–9 g per 100 mL water is recommended by the International Standard for the preparation of coffee.^19^ Samples were extracted (brewed) for 3 min while being stirred on a magnetic mixer. After the mixture had been brewed, solids were removed by filtering through Miracloth (Calbiochem, San Diego, USA) in a Buchner funnel. Brewed samples were collected in thermos bottles to keep them warm for sensory evaluation.

Three different roasting batches of coffee cells were as described above (section 2.2. Roasting of Coffee Cells) and were used. Two diluted, conventional coffee references were prepared with dilution factors based on the selection of the descriptive sensory panel. First, the dark roast Pelican Rouge sample was prepared by brewing 6 g of coffee grinds with 100 mL of water as above and then mixing 50 mL of additional water after brewing (dilution to 67%). Second, the light roast Juhla Mokka sample was likewise brewed with 6 g of grounds, 100 mL of water ratio, with 20 mL additional water added after brewing (dilution to 83%). Additionally, instant chicory coffee “Chikko not coffee” (Ghee Easy B.V., Amsterdam, Netherlands) was prepared at 5 g per 100 mL of water (according to the manufacturer’s instructions).

### 2.8 Color Measurement

The color of each brewed sample and filter cake, as well as the two commercial control coffees (Pelican Rouge Rich Blend and Juhla Mokka), was measured with a Minolta chroma meter CR-200 (Minolta Camera CO., Ltd., Japan). The instrument was calibrated with a white ceramic plate (Calibration plate CR-A43). For liquid samples, 3 mL of sample was pipetted into a small Petri dish (Ø = 3 cm) and the Petri dish was covered with white paper. The color measurements were made from each brew used in the training and evaluation sets in the sensory profiling for a total of four replicate brews. The first batch of the liquid samples for the roast 2 sample was removed from the data set due to a failed brew (lighter color, measurement values deviating >3 standard deviations). Color values were recorded from the bottom of the Petri dish at five different points each. For filter cake samples, a small Petri dish (Ø = 3 cm) was filled with filter cake and otherwise, the same procedure as with liquid samples was used. Three mL of RO-water was used as a blank control sample. The color was recorded as coordinates in the CIE Lab color space, where *L* = 0 is black, *L* = 100 white, −a = green, +a = red, −b = blue, and +b = yellow. The total color difference (Δ*E*) between each sample and control water sample was defined by the following equation:
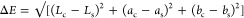
where *c* refers to the control water sample and *s* to the other samples.

### 2.9 Sensory Profiling

The sensory profiles of coffee samples were analyzed by 8 assessors of VTT’s trained food and beverage sensory panel using generic descriptive analysis. An application regarding the sensory evaluation was submitted to VTT’s internal ethical committee. The risk mitigation strategies for the panel included following a taste-and-spit assay, ensuring the microbiological quality of the samples, using small evaluation volumes, complying with COVID-19 precautions, and requesting prior written informed consent from the assessors.

The base lexicon for the coffee samples was formulated by four panel members in a consensus tasting session by cross-referencing the samples with published sensory lexicons and suggested reference products for coffee.^20−22^ In the same session, the appropriate dilution for conventional coffee (section 2.2) was selected to minimize intensity contrast effects. This was done by offering coded samples in different dilutions and selecting the one closest in total flavor intensity in relation to cell coffee samples. This base lexicon was trained and refined with the whole panel (divided in two groups), and the reference product intensities were tied to the 0–10 line scale. The resulting sensory lexicon had seven odor attributes and five taste or flavor attributes, which were tied with 10 reference products (see Table S3 in the Supporting Information for the list of attributes and the reference products). The sensory evaluation was done in VTT’s ISO-8589 sensory evaluation laboratory. The samples were presented monadically in a balanced complete block design using Latin squares serving order randomization. For each sample, 20 mL of the liquid was poured from the thermos bottles to 60 mL beakers (both marked with corresponding 3-digit codes), and a plastic lid was placed over the beakers for 2 min before starting the evaluation. Two repeat evaluations were made. The sensory data was collected using EyeQuestion version 5.0.7.15 (EyeOpenR Data Analysis) by EyeQuestion Software (Elst, The Netherlands) and Qi Statistic Ltd. (West Malling, UK).

### 2.10 Gas chromatography–Olfactometry Analysis

The headspace solid-phase microextraction-gas chromatography–mass spectrometry/olfactometry (HS-SPME-GC-MS/O) analysis of the coffee samples was adapted from the protocols of Akiyama et al.^15^ and López-Galilea et al.^16^ with some modifications. Dried unroasted and roasted coffee cells were stored in darkness at ambient temperature in sealed foil bags before extraction. All samples were prepared within 1 h before the GC-O analysis following the procedure described in section 2.3. Volatile compounds were extracted by SPME with a 2 cm 50/30 μm DVB/CAR/PDMS fiber (Stableflex, 23Ga by Supelco, Bellafonte, PA).^16^ After brewing, 1.0 mL of the freshly brewed cell coffee sample was transferred into a 20 mL screw-cap vial equipped with a poly(tetrafluoroethylene)/silicone septum (Supelco, Bellafonte, PA). The sample vials were incubated using the autosampler (Combi PAL, PAL System, CTC Analytics AG, Zwingen, Sitzerland) for 1 min at 60 °C and with stirring at 250 rpm. The SPME fiber was inserted into the vial, and the fiber was exposed to the headspace above the coffee sample for 30 min at 60 °C to extract the volatile compounds. The fiber was desorbed for 6 min in splitless mode (split opened after 1.5 min) in the injection port at 250 °C of the GC-MS/O system, which consisted of a 6890N GC (Agilent Technologies, CA, US) equipped with a mass detector (5973-Network) and a sniffing port, ODP4 (Gerstel, Baltimore, MD). The flow rate of the helium carrier gas was set to 2.0 mL/min. The volatiles were separated in a VF-WAXms capillary column (60 m × 0.25 mm × 0.5 μm, Agilent Technologies, CA, US). The temperature program of the oven was the following: hold at 50 °C for 1 min, from 50 to 150 °C at 15 °C min^–1^, then to 240 °C at 8 °C min^–1^, and hold at 240 °C for 5 min. The GC effluent was split 1:1 between the mass detector and sniffing port, which was supplied with humidified air at 40 °C. A quadrupole mass selective detector, with electronic impact ionization (ionization energy = 70 eV) operated in scan mode, has a mass range of 25–600 amu, at 2.0 scans/s. Temperature of the MS detector was set at 230 °C. The volatile compounds were analyzed from an average of four replicate chromatograms, calculated, and expressed as the area percentage of their abundance (total area %). The initial identifications were confirmed with a secondary BPX-5 column (60 m × 0.25 mm × 0.5 μ, SGE Analytical Science Pty Ltd., Victoria, Australia). The extraction and MS conditions were the same as those with the primary column. The GC-MS system used for the analysis of volatile compounds without olfactometry consisted of a 7890B GC instrument (Agilent Technologies, CA, US) equipped with a mass detector (5977B). The following oven temperature profile was used: hold at 50 °C for 1 min, from 50 to 170 °C at 10 °C min^–1^, then to 260 °C at 15 °C min^–1^ and hold at 260 °C for 11 min. Also, the GC-MS/O analysis of three samples was replicated utilizing the BPX-5 column with the same instrumentation as that with the VF-Wax column. The following temperature profile was used: hold at 60 °C for 1 min, from 60 to 130 °C at 7 °C min^–1^, then to 230 °C at 15 °C min^–1^, and to 300 °C at 15 min^–1^ and hold at 300 °C for 3 min.

GC-O evaluation was performed with the detection frequency (DF) method with a panel of four assessors (three females and one male). Additionally, the tentative GC-O observations on the primary column were confirmed by two assessors with the secondary GC column. All panelists were previously trained in odor recognition and sensory evaluation techniques and had experience in GC-O. The panelists were asked to describe the odor and record the duration of each odorant. Detection of an odor at the sniffing port by three or more assessors was considered significant.

The volatiles were tentatively identified based on (a) NIST library (vs2.3, 2017), (b) linear retention indices from two columns were calculated based on a hydrocarbon standard mixture (C7–C30 saturated alkanes, Supelco, Bellafonte, PA), (c) by analyzing authentic standards with the same extraction and gas chromatography protocol as the samples, and (d) by comparison to the previously published literature^15−17,23−25^ for typically identified compounds and their odor properties. The following reference compounds were purchased from the suppliers given in parentheses: 2,3-butanedione, 2,3-pentanedione, hexanal, decanal, benzaldehyde, 2-methoxyphenol and 2-phenylacetaldehyde (Sigma-Aldrich, Saint Louis, Missouri, US), and 3-hydroxy-2-butanone (Fluka, North Carolina, US). A semiquantification, that is, peak normalization based on relative intensities, was performed on the BPX5 column with 3-octanol (Sigma-Aldrich, Saint Louis, Missouri, US) as an internal standard for the additional analyses. The contents were calculated as 3-octanol equivalents, assuming a response factor of 1 for all compounds to allow comparison between samples.

### 2.11 Statistical Analysis

For color measurement, an average of the five measurement points was used, and the sample average and standard deviations were calculated based on differences between the four batches. A one-way ANOVA (or the robust Brown-Forsythe test) with Tamhane’s or Tukey’s post hoc tests (depending on the equivalence of variances) was used to examine the statistically significant differences between samples.

The sensory evaluation data were analyzed with a two-way mixed model analysis of variance. The samples were used as a fixed factor and the assessors as a random factor, and the product × assessor interaction was included in the model. Tukey’s HSD was used as the post hoc test. This testing was done using IBM SPSS version 26 (IBM Corp, New York, USA). The limit of statistical significance was set as *p* < 0.05. The color measurements and sensory profiles were visualized using principal component analysis (PCA) with the Unscrambler version 10.5.1 (CAMO Software AS, Norway) using averaged, autoscaled data.

## 3 Results and Discussion

### 3.1 Characterization of Coffee Samples

#### 3.1.1 Color and Appearance of the Coffee Samples

**Figure 1:**
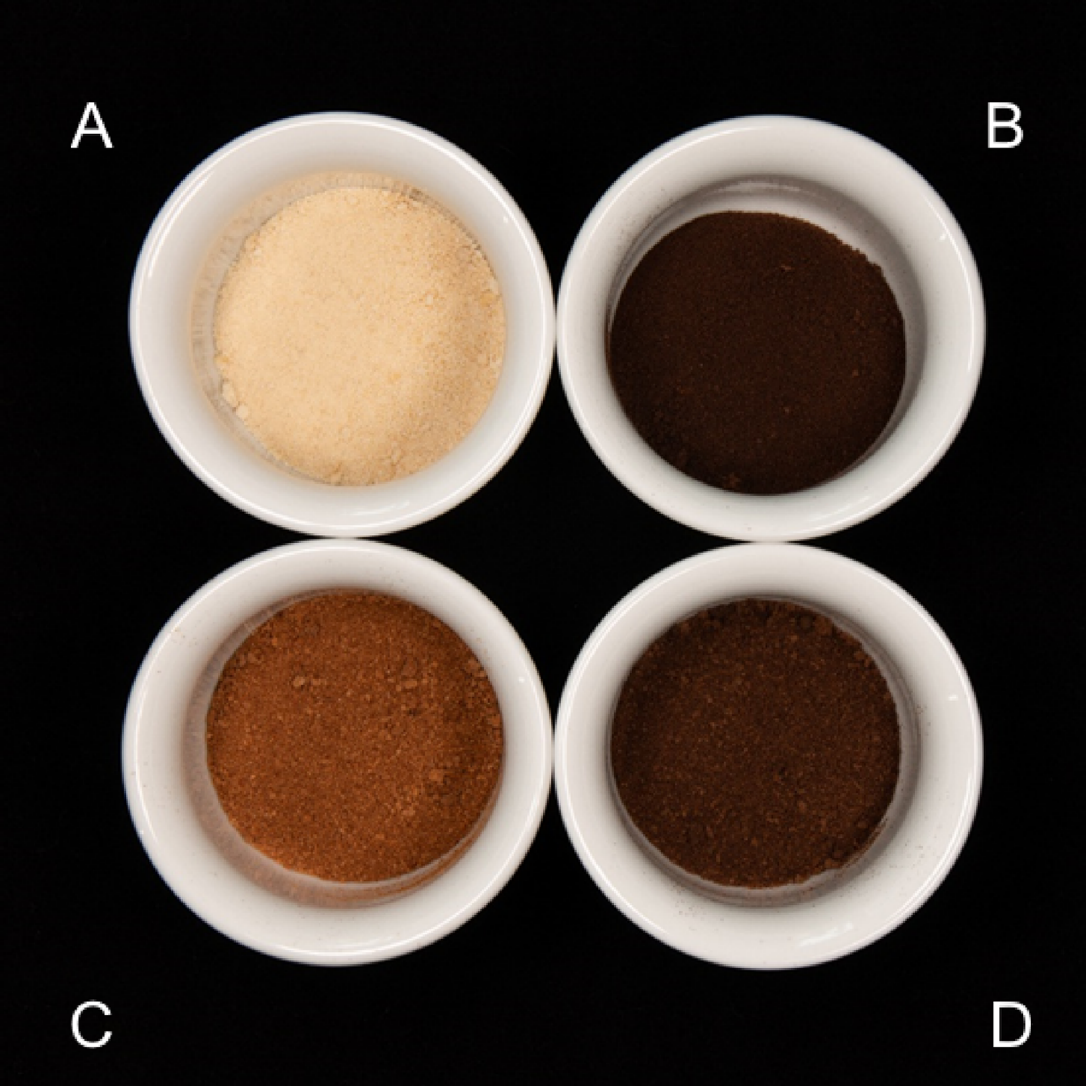
Coffee cell samples: (A) Unroasted, lyophilized coffee cells (L* = 48.8); (B) roast 1 (L* = 31.0); (C) roast 2 (L* = 36.0); (D) roast 3 (L* = 32.0) (roasting conditions described in section 2.2). L* = lightness value (dark–light, 0–100) according to the color spacer defined by the International Commission of Illumination (CIE).

Cell cultures in this study were initiated from *Coffea arabica* leaves following initially the protocol of Teixeira et al.^18^ Other methods such as using explants from cotyledons and using rather rich media have been reported earlier.^26^ Although Townsley,^10^ who reported the use of plant cell cultures for coffee for the first time, used stem sections and employed different growth media compositions, the resulting cell cultures appear very similar to “a light cream color becoming darker as the incubation period is increased”. The lyophilized cells derived from the bioreactor cultivations were beige (Figure 1A). Different roasting regimes turned the material from light to very dark brown (Figure 1B–D) similar to the color of commercial ground coffee. Due to the importance of the visual aspects of both the raw material^27,28^ and the brew,^29^ an objective color measurement provides an indication how close the cell-cultured version is to a commercial reference. Conversely, Wang et al.^30^ demonstrated that altering the color of coffee in a virtual reality environment can affect the perceived flavor, which also indicates the importance of reaching similar color properties for cell-based coffee as conventional coffee.

The effect of the roasting was indicated instrumentally in the L*a*b* values of the color measurements, which are overviewed in Table 1 and in the Supporting Information (Figures S1 and S2). In the solid samples, roasting decreased the L* values (darker samples) and especially the b* values (blue/yellow axis), while the change in a* values (red/green axis) depended on the roasting parameters. Roast 2 was the mildest treatment based on the values as the L*a*b* (36.0, 5.6, 6.6) values were the closest to unroasted cells (48.8, 5.6, 13.9) among the three roasting conditions. These values were 2–3 units larger than with the light roasted control coffee (Juhla Mokka, 33.1, 3.8, 3.7). On the other hand, roast 3 was very similar to the dark roasted control coffee (<1 unit difference in the L*a*b values, while roast 1 had even smaller values than the dark roasted coffee).

**Table 1:** Color Measurements of the Liquid and Solid Samples^a^

**sample**	L*			a*			b*			**Δ***E*		
ANOVA (*p*)	<0.001			<0.001^b^			<0.001			<0.001		
**solid samples**												
light roast Juhla Mokka	33.1	(0.6)	c	3.8	(0.4)	b	3.7	(0.6)	c	28.7	(0.8)	b
dark roast Pelican rouge	32.8	(0.5)	c	2.1	(0.1)	c	1.6	(0.2)	d	28.5	(0.5)	b
unroasted coffee cells	48.8	(0.4)	a	5.6	(0.3)	a	13.9	(0.7)	a	20.2	(0.5)	d
roasted coffee cells, roast 1	31.0	(0.7)	d	1.6	(0.2)	c	0.9	(0.2)	d	30.2	(0.7)	a
roasted coffee cells, roast 2	36.0	(0.6)	b	5.6	(0.6)	a	6.6	(0.6)	b	26.9	(0.3)	c
roasted coffee cells, roast 3	32.0	(0.9)	cd	2.4	(0.3)	c	1.5	(0.2)	d	29.4	(0.9)	ab
**liquid samples**												
ANOVA (*p*)	<0.001^b^			<0.001			<0.001			<0.001		
Juhla Mokka (diluted)	45.9	(1.3)	bc	4.2	(0.2)	c	26.0	(0.9)	c	31.4	(0.3)	b
dark roast Pelican rouge (diluted)	43.4	(1.9)	bc	7.5	(0.7)	b	25.1	(1.6)	c	32.6	(0.4)	b
unroasted coffee cells	54.1	(0.6)	a	2.1	(0.3)	d	9.6	(0.7)	d	13.0	(0.9)	c
roasted coffee cells, roast 1	47.9	(0.4)	b	6.9	(0.6)	b	32.7	(0.3)	a	36.9	(0.5)	a
roasted coffee cells, roast 2^c^	44.2	(0.2)	c	9.4	(0.5)	a	30.3	(0.2)	b	36.9	(0.4)	a
roasted coffee cells, roast 3	44.4	(0.5)	c	10.2	(0.7)	a	30.9	(0.7)	ab	37.5	(0.6)	a

a The table shows the average values, the standard deviations (in parentheses), and ANOVA post hoc groups. Samples with a different letter have statistically significant different values in each variable.

b Based on Brown-Forsythe, Tamhane T2 as post hoc.

c From three batches due to the first failed brew.

Comparing to the previously published literature on conventional coffee beans, unroasted coffee cells had similar L* values (1.1 unit difference) as those reported by Kim et al.^28^ However, the a* and b* values were 3 units larger. On the other extreme, both conventional coffee grounds as well as the roasted coffee cells in the present study were in the same range as light roasted samples reported by Yeager et al.^29^ Furthermore, different roasting regimes showed similar differences in L*, a*, and b* values in ground green beans (Supporting Information Table S4).

Similar changes were observed in the beverages made from the cells. Roasting decreased the L* values (darker samples) and increased the a* (redder samples) and b* values (yellower samples). The liquid samples were not a complete match with the (diluted) conventional coffee beverages. While roast 2 and 3 had similar L* values to the conventional coffees and roast 1 had a similar a* value, all three roasted samples had higher b* values than the conventional coffee. This indicates that the extraction yield was smaller with the cell-based coffees than with conventional samples.

Previously, it has been reported that the different degrees of brown color in roasted coffees are linked to the different classes of melanoidins as well as differences in sugar contents and profiles.^13,29^ Furthermore, changing the sugar contents of green coffee beans has been previously reported to influence the color of the treated, roasted coffee beans.^31^ While the sugar and amino acid contents of the coffee cells were not analyzed in the present study, a range between 18 and 38 mg/g Dw free sugars and 11.8 to 22.9% Dw of amino acids has been reported earlier in plant cell cultures.^32,33^ This would allow for sufficient Maillard reaction precursors in the coffee cells.

#### 3.1.2 Caffeine Content and Toxicity

The stimulating property of coffee is due to the purine alkaloid caffeine (1,3,7-*N*-trimethylxanthine), but the compound is partly responsible for the bitterness of the beverage,^12^ too. Previously, rather low levels of caffeine (0.09 mg/g DW) have been reported in coffee cell cultures grown under light and complete absence has been noted in cells cultivated in darkness.^34^ In contrast, this study finds a slightly higher caffeine content (0.22 mg/g DW) even in dark-grown cells (Table 2). Although this amount is far from the level reached in coffee beans (Table 2) and most likely does not carry any sensory relevance, it nevertheless shows that the biosynthetic pathway is active in the cell culture. The biosynthesis proceeds from xanthosine via three consecutive methylations to caffeine. There is evidence that AlCl_3_ addition activates key enzymes leading to higher caffeine accumulation.^34^ Elicitation such as the described Al-addition or with other stressors could be a powerful tool for future optimization of cell culture conditions toward the production of relevant secondary metabolites including caffeine.

**Table 2:** Caffeine Content and Acute Toxicity Assessment with *Daphnia magna* of the Coffee Cell Culture and Coffee Bean Samples

			EC_50_ values [mg/L]	
samples	caffeine [mg/g]	pH	24 h	48 h
lyophilized coffee cells	0.22	6.96	>1000	>1000
lyophilized and roasted coffee cells	0.22	6.81	>1000	>1000
roasted Arabica beans	8.59	6.96	930	680
K_2_Cr_2_O_7_ (negative control)			0.83	0.93

Coffee consumption has a long history of safe use. Both green coffee beans and roasted coffee are considered safe based on acute toxicity tests in rats at a dose of 2000 mg/kg.^35,36^ In vivo studies with mammals are costly and have not yet been reported for the evaluation of plant cell-derived food. *Daphnia magna*, a freshwater crustacean, is however widely used to assess acute toxicity of compounds or extracts. The EC50 value defining the concentration of the test sample in which 50% of *Daphnia* neonates die and/or are immobilized during 24 and 48 h of incubation is calculated in a standardized way.^37^ In this test, roasted Arabica coffee beans exhibit a slightly stronger effect on the survival of *Daphnia* than the coffee cells (Table 2). Toxicity of pure caffeine for *Daphnia* had been observed with a > 90% mortality after exposure to 800 mg/L of caffeine,^38^ and the higher concentration in the beans could possibly explain the difference. The pH values of all the samples are in a similar, slightly acidic, range and could influence the results on a general level because optimum conditions for *Daphnia magna* range from pH 7.9 to pH 8.3.^39^ Furthermore, the sensitivity of this organism to dark-colored liquids has been noticed previously.^40^*Daphnia* are sensitive to phenolics such as caffeic acid, too.^41^ However, the investigated samples exhibit toxicity values close to previously evaluated plant cell samples.^33^

The microbial load of the samples was below the threshold value (10 CFU/g or less than 1 CFU/mL), and therefore, the samples were approved for sensory evaluation.

### 3.2 Sensory Properties of Brewed Cell Coffee

**Figure 2:**
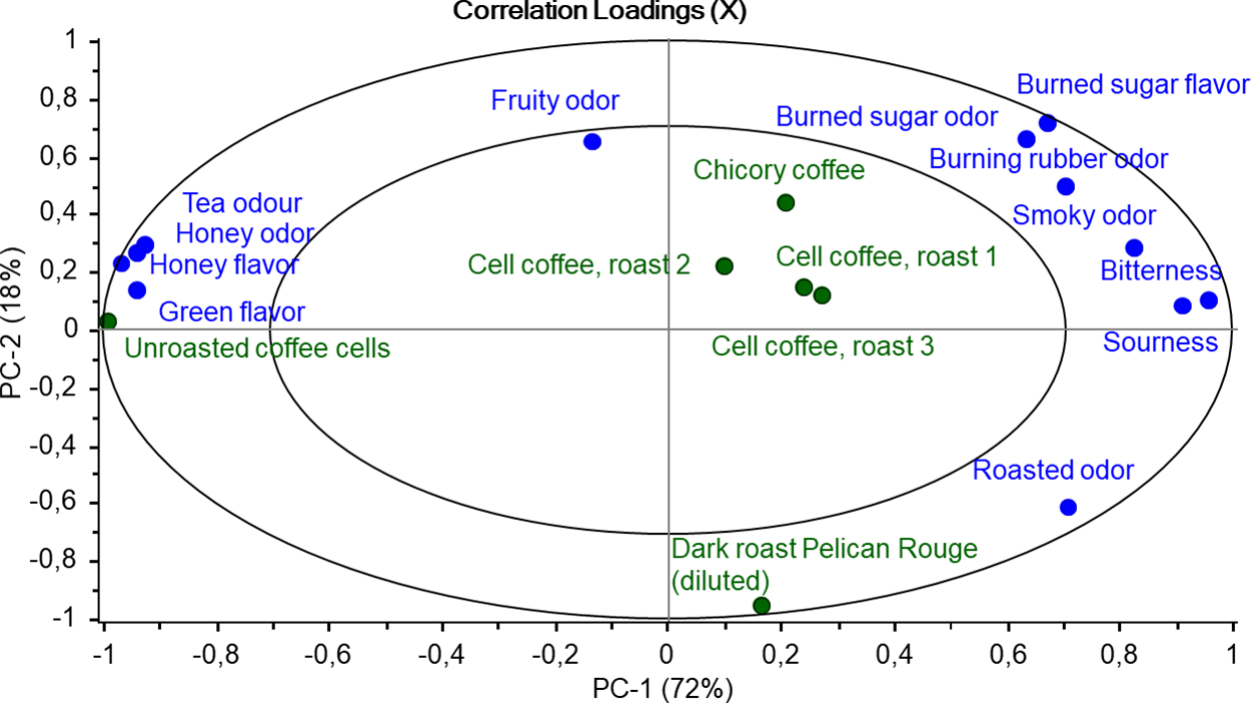
Principal component analysis correlation loadings plot of the sensory properties of the coffee samples.

The sensory profiles of six beverages brewed from unroasted coffee cells, three different roasting parameters for coffee cells, diluted dark-roasted conventional coffee, and chicory coffee alternative were determined with generic descriptive analysis. The roasting of coffee cells with different parameters changed multiple sensory attributes of the brewed samples toward conventional coffee (Table 3, Figure 2, and Supporting Information Figure S3). Samples brewed from unroasted coffee cells lacked most of the typical attributes for coffee, such as roasted odor, sourness, and bitterness. Instead, the samples were perceived to be the most intensely honey- and tea-like. These odor properties were still minimally present in roast level 2, but in roast levels 1 and 3 the dominant odor attributes were instead roasted, burned sugar, and smoky odor. These changes in sensory aroma characteristics largely aligned with those reported in conventional coffee counterparts:^42^ the earthy/musty and green attributes in the published literature were lost in favor of coffee, roasted, burnt/acrid, and ash/sooty attributes with darker roast levels. For taste attributes, the roasted cell coffees had comparable intensities of bitterness (cell coffees 5.5–6.5 vs 0.1% caffeine solution at 7) and sourness (cell coffees 6–8 vs light roasted coffee reference at 9) to conventional coffee.

**Table 3:** Sensory Characteristics of the Cell Coffee Samples^a^

sample	ANOVA (*p*)	partial η^2^	diluted dark roast Pelican Rouge			unroasted coffee cells			cell coffee, roast 1			cell coffee, roast 2			cell coffee, roast 3			chicory coffee		
roasted odor	<0.001	0.591	5.8	(1.6)	a	0.9	(0.9)	c	4.0	(2.6)	b	1.9	(1.8)	c	3.9	(1.9)	b	3.2	(2.1)	b
burned sugar odor	<0.001	0.489	1.3	(1.4)	b	1.3	(1.2)	b	4.1	(2.3)	a	2.5	(1.1)	b	4.2	(2.2)	a	3.9	(3.1)	a
smoky odor	<0.001	0.514	2.0	(2.2)	b	0.5	(0.7)	c	4.2	(2.7)	a	2.0	(2.1)	b	3.7	(2.4)	a	3.1	(2.6)	ab
burning rubber odor	0.062	0.251	0.9	(1.2)		0.6	(0.8)		2.5	(2.4)		1.3	(1.5)		2.3	(2.4)		1.9	(2.0)	
tea odor	<0.001	0.697	0.7	(0.9)	d	6.0	(1.5)	a	1.9	(2.1)	bcd	2.8	(1.8)	b	2.1	(2.2)	bc	1.6	(1.6)	cd
honey odor	<0.001	0.743	0.6	(0.7)	c	5.7	(1.3)	a	1.2	(1.6)	bc	2.2	(1.7)	b	1.3	(1.4)	bc	1.4	(1.6)	bc
fruity odor	0.058	0.254	0.4	(0.4)		1.4	(1.9)		0.5	(0.7)		1.8	(2.2)		0.8	(0.7)		2.6	(2.8)	
sourness	<0.001	0.740	6.3	(2.3)	b	2.0	(1.7)	c	6.1	(2.5)	b	8.0	(1.0)	a	7.0	(2.1)	ab	6.5	(2.3)	b
bitterness	<0.001	0.731	5.3	(2.5)	a	1.6	(1.4)	b	5.9	(2.6)	a	6.5	(1.7)	a	6.2	(2.5)	a	5.5	(2.4)	a
burned sugar flavor	<0.001	0.640	1.7	(1.7)	c	1.3	(1.1)	c	4.9	(3.0)	ab	4.4	(2.5)	b	4.6	(1.9)	b	6.2	(1.9)	a
honey flavor	<0.001	0.818	0.4	(0.5)	c	5.1	(1.2)	a	1.5	(1.9)	bc	1.0	(1.0)	bc	0.9	(0.9)	bc	1.8	(1.8)	b
green flavor	<0.001	0.484	0.5	(0.5)	b	2.0	(1.4)	a	0.9	(1.2)	b	1.0	(1.1)	b	0.4	(0.6)	b	0.5	(0.7)	b

a The table contains the averages, standard deviations (in brackets), two-way mixed model ANOVA *p*-values, and effect size estimates as well as Tukey’s HSD post hoc groups. The products in each attribute marked with a different letter (a–d) have statistically significant differences.

As discussed in section 3.1.2, the cell coffees had only low levels of caffeine, which did not contribute to bitterness as demonstrated by the low bitterness intensity (1.6) in unroasted coffee cells. However, the bitterness in coffee is mainly formed in the roasting process,^43^ with the degradation products of chlorogenic acid derivatives as the main contributors.^44,45^ Recently, Lang et al.^12^ examined the potency of bitter compounds found in coffee via TAS2R receptor activation and sensory tests and demonstrated that various compounds such as mozambioside, cafestol, and kahweol can contribute more to bitterness than caffeine. Different carboxylic and caffeoylquinic acids contribute to sourness,^13,46^ with the aliphatic acids content increasing^47^ and the chlorogenic acid content decreasing during roasting.^46^ A similar phenomenon was indicated in the sensory profile of the present study, where a lower sourness was observed in the lighter roast 2 sample.

Analysis of major quinic acid esters and phenolic acids indeed showed a clear decrease of caffeoylquinic acid and an increase of caffeoylquinic acid caused by roasting (Table 4). Generally, the concentrations of the individual compounds in coffee cells and coffee beans varied but were mostly on a similar level except for dicaffeoylquinic acid (Table 4).

**Table 4:** Major Caffeoylquinic and Phenolic Acid Contents in Coffee Cell Culture and Green Coffee Bean Samples^a^

	**CQA**	**FQA**	***p*-CoQA**	**di-CQA**	**caffeic**	**ferulic**	***p*-coumaric**
samples	[mg/g]	[mg/g]	[mg/g]	[mg/g]	[mg/g]	[mg/g]	[mg/g]
lyophilized coffee cells	204	21	5	39	9.8	1.0	1.8
lyophilized and roasted coffee cells	157	27	3	141	9.2	0.8	1.6
green coffee beans	240	51	1	181	10.0	2.5	0.3

a CQA: caffeoylquinic acid, FQA: feruloylquinic acid, *p*-CoQA: *p*-coumaroylquinic acid, and di-CQA: dicaffeoylquinic acid.

Finally, it should be noted that the method of coffee preparation has been shown to strongly influence the sensory attributes of the resulting brew such as roasted odor, other aroma attributes, sourness, and bitterness.^11,23,48,49^ In this study, the cell coffees were compared to diluted dark roast coffee and all samples were brewed as filtered coffee. Future work should also account for the differences in the roasted biomass such as particle size and adapt the brewing process accordingly.

### 3.3 Odor-Active Volatiles of Cell Coffee

The odor-active volatiles of the conventional coffee and cell coffee samples were tentatively identified with the detection frequency method by GC-O with four trained panelists. Twenty-six odor-active compounds with a nasal impact factor (NIF) equal or above 50% were detected in the conventional coffee, 22 were detected in the unroasted cell coffee samples, 22 were detected in the roast 1 cell coffee samples, and 21 were detected both in the roast 2 and roast 3 cell coffee samples (Table 5). Identification was obtained by comparing the calculated LRI values, mass spectra, and odor descriptors to those of pure compounds. The relative peak areas are presented in Supporting Information Table S5 and semiquantification of the volatile compounds using BPX5 column in Supporting Information Table S6.

**Table 5:** Odor-Active Volatiles of Cell Coffee and Conventional Coffee Samples^a^

			LRI^b^	LRI^b^	NIF^c^ (%)					
no	compound	identification	VF-WAX	BPX5	UC	R1	R2	R3	PR	odor description^e^
1	2,3-butanedione	O, MS, RI^f^, RI2^g^, std^h^	987	599	25	75	50	nd^d^	75	cacao, caramel, chocolate, vanilla
2	2,3-pentanedione	O, MS, RI, RI2	1076	702	75	25	25	nd	25	caramel, apple, sweet
3	hexanal	O, MS, RI, RI2, std	1111	811	75	75	50	50	nd	grass, green
4	(*E*)-2-methylbut-2-enal (tentative)	MS, RI	1135		nd	nd	nd	nd	100	smelly
5	(*Z*)-hept-4-enal	O, MS, RI	1272		50	75	75	75	50	wool, chocolate, green
6	2-methylpyrazine	O, MS, RI, RI2	1309	845	nd	nd	nd	nd	100	pungent, solvent, nail polish
7	1-octen-3-one	O, MS, RI, RI2	1332	976	100	50	50	100	nd	mushroom
8	3-hydroxybutan-2-one	MS, RI, RI2, std	1331		nd	nd	nd	nd	75	green, mushroom, grass
9	2,5-dimethylpyrazine	O, MS, RI, RI2	1364	935	nd	nd	nd	nd	75	butter, medicine
10	(*E*)-hept-2-enal	O, MS, RI	1365		50	25	75	50	nd	beany, meat broth
11	unknown		1388		25	25	25	25	50	roasted, peanut
12	2-ethyl-6-methylpyrazine	O, MS, RI, RI2	1424	1020	nd	nd	nd	nd	100	sweet, essence, candy
13	2-ethyl-5-methylpyrazine	MS, RI, RI2	1433	1025	50	50	nd	50	25	sweet, chocolate, caramel
14	2,3,5-trimethylpyrazine	O, MS, RI	1444		nd	nd	nd	nd	50	forest, bean
15	2-ethyl-3-methylpyrazine	O, MS, RI	1444		nd	nd	nd	nd	50	forest, bean
16	unknown		1450		nd	nd	nd	nd	100	bean, spice, unpleasant
17	heptan-1-ol	O, MS, RI, RI2	1365	974	25	nd	50	25	nd	fruit, mushroom, biowaste, grass
18	2-propylpyrazine	O, MS, RI	1462		nd	nd	nd	nd	75	biowaste, mushroom, compost, grass
19	unknown		1471		75	50	nd	nd	nd	play dough, bread, pencil
20	unknown		1474		50	nd	50	nd	100	grass, bitter, plant
21	unknown		1479		nd	50	75	100	nd	coffee, roasted
22	unknown		1500		nd	nd	75	25	100	mold, raw bean, sprouts
23	(*2E*,*4E*)-hepta-2,4-dienal	O, MS, RI	1505		75	nd	nd	nd	nd	potato, sweet potato, rye
24	1-(1-methoxypropan-2-yloxy)propan-2-ol (tentative)	MS, RI	1504		nd	75	50	75	nd	raw bean, roasted, solvent, acidic
25	3,5-diethyl-2-methylpyrazine	O, MS, RI, RI2	1528	1175	nd	nd	nd	nd	75	mold, grass, dirt
26	unknown	O, MS, RI	1524		nd	100	25	25	nd	raw bean, plant, grass
27	decanal	O, MS, RI, RI2, std	1527	1214	50	nd	nd	nd	nd	paint, musty
28	furan-2-ylmethyl acetate	O, MS, RI, RI2	1559	1003	50	75	50	50	100	grass, green, raw bean
29	unknown		1573		75	nd	25	nd	nd	pungent, plant
30	benzaldehyde	MS, RI, RI2, std	1588	1001	100	100	25	50	nd	wooden, glue, wall
31	unknown		1604		25	25	50	nd	75	grass, forest, plant, mold
32	unknown		1632		50	100	100	100	75	green, grass, hay, cucumber, plant
33	unknown	O, MS, RI	1687		nd	nd	nd	nd	75	popcorn, salted peanut
34	2-phenylacetaldehyde	O, MS, RI, RI2, std	1704	1080	100	50	75	75	100	honey, floral, soap, red berry
35	unknown		1730		50	100	25	25	nd	cooked bean, meat broth, roasted
36	unknown		1753		50	50	50	75	nd	plastic, bitter, play dough
37	unknown		1860		50	50	25	75	25	plant, soap, bitter, play dough
38	unknown		1877		75	25	50	25	75	rowan berry, rose, fruity
39	unknown		1888		nd	50	nd	50	50	roasted sugar, caramel, sweet
40	2-methoxyphenol (guaiacol)	O, MS, RI, RI2, std	1912	1119	nd	nd	nd	nd	75	chemical, pungent
41	unknown		1919		nd	75	50	50	25	cotton candy, sweet
42	unknown		1955		nd	nd	nd	25	75	fish, essence, cherry
43	3-hydroxy-2-methylpyran-4-one (maltol)	O, MS, RI, RI2, std	2027	1149	75	100	50	75	75	caramel, sweet, oat cookie
44	unknown		2041		75	50	25	50	25	musty, paint, unpleasant
45	pentadecanal	O, MS, RI	2055		75	nd	nd	nd	nd	nature wood earthy, green
46	4-hydroxy-2,5-dimethylfuran-3-one (Furaneol)	O, MS, RI, RI2	2075	1068	nd	100	100	100	100	cotton candy, sweet, roasted sugar
47	unknown		2087		nd	25	50	75	nd	roasted, musty
48	unknown		2101		nd	nd	50	100	nd	sweet, strawberry, vanilla, candy
49	unknown		2123		nd	nd	nd	nd	100	candy, sweet, roasted

a UC = unroasted cell coffee, R1= roast level 1 cell coffee, R2= roast level 2 cell coffee, R3 = roast level 3 cell coffee, PR = dark roast Pelican Rouge, conventional Arabica coffee.

b Linear retention index.

c Nasal impact factor.

d Not detected.

e Odor descriptions provided by the GC-O panelist.

f Identification based on retention index determined by VF-457 Wax column.

g Identification based on retention index determined by BPX5 column.

h Identification based on the commercial pure compound standard.

In general, the unroasted cell samples had mainly odor-active compounds described as green, grass, and beany such as hexanal, (*Z*)-4-heptenal, 2,4-heptadienal, and pentadecanal. In the previous literature, the main odor-active compounds of green coffee beans were reported to be 2-methoxy-3,5-dimethylpyrazine (earthy), hexanal (green), ethyl-2- and −3-methylbutyrate (fruity), 3-isobutyl-2-methoxypyrazine (pea-like), and 4-ethylguaiacol (sweet).^17^ The different pyrazines had comparatively high flavor dilution (FD) and odor activity values (OAVs) compared to other reported odor-active compounds.^17^ While unroasted coffee cells in the present study shared many of the odor percepts as green coffee beans, the underlying odor-active compounds were different. Clearly, and as expected, the biosynthesis is differentially regulated in organs with diverse specialized tissues such as coffee seeds and in undifferentiated cells in coffee cell cultures, reflecting the composition of accumulated compounds.

Roasting significantly affected the odor-active compounds of the cell coffee samples. Most of the green- and grass-type odors were either not detected in the roasted samples or their NIF values and relative abundances decreased. Many of the odors described as plant-like, green, beany, and mushroom-like were observed only in cell coffee samples while not at all in the conventional coffee such as 1-octen-3-one, (*E*)-2-heptenal, and 1-heptanol. Most of these compounds have not been described in the earlier literature as coffee odorants.^11,15−17,25,26^ These findings show that there are some critical differences between the odor profiles of conventional coffee and current cell coffee samples.

For conventional coffee, López-Galilea et al.^16^ reviewed and later, for example, Laukaleja et al.^23^ reported how the formation of pyrazines, pyridines, pyrroles, and furans is related to Maillard reaction between reducing proteins and carbohydrates that are naturally present in green coffee beans. For example, Czerny and Grosch^17^ demonstrated how the contents of 3-hydroxy-4,5-dimethyl-2(5*H*)furanone increased over 1000-fold due to roasting. Similar expected changes were also seen in roasted cell coffee samples, where several new odor-active compounds were formed compared to unroasted cells. This change in relevant volatiles is demonstrated in Figure S5: the stronger roasted cell coffees become more similar in their volatile composition to conventional coffee (positive loading in principal component 1). A similar trend was seen for roasted green beans, although the change was less pronounced.

Looking at individual compounds, 2-ethyl-5-methylpyrazine described as “sweet, chocolate, caramel” was observed in both roasted cell coffee and conventional coffee samples. On the other hand, several other pyrazines such as 2,5-dimethylpyrazine, 2-ethyl-6-methylpyrazine, 2,3,5-trimethyl pyrazine, and 2-ethyl-3-methylpyrazine were observed in the conventional coffee but not in the roasted cell coffees. Likewise, many of the sweet and caramel like odors were observed in both roasted cell coffee samples and in the conventional coffee such as 2,3-butanedione, 2,3-pentanedione, maltol, and furaneol. These compounds have been commonly reported in conventional coffees and are considered important for the coffee aroma.^11,15,16,25^ Similarly, 2-phenylacetaldehyde described as “honey, floral, soap, red berry” was observed in all cell coffee samples and has been previously reported to be an odor-active compound in coffee,^23^ although it was only detected by GC−MS in the conventional coffee sample. 2-Methoxyphenol (guaiacol) is considered an important coffee odorant as reviewed by López-Galilea et al.^16^ and was only observed in the conventional coffee sample in the current study. These findings show that the cell coffee samples lacked some important coffee odorants, but many important ones were present. Likely, the simple roasting process utilized in the current study resulted in some different odor-active Maillard reaction products in the cell coffee samples, which explains the differences observed by the sensory panel between the cell coffee samples and the conventional coffee.

Several odor-active compounds were observed by the panel both in the conventional coffee sample and in cell coffee samples that were not identified based on the mass spectrum and are referred as unknown compounds. These included the green and grass-like compound at RI 1632 and the berry, rose, and fruit-like compound at RI 1877. On the other hand, an unknown compound at RI 1479 described as coffee and roasted-like was observed in all three roasted cell coffees but not in the unroasted cells or conventional coffee sample. This result further highlights that the roasted odor of the cell coffee samples originated from different compounds than that in conventional coffee, which is also supported by the observations from the sensory analysis.

It is evident based on the current study and literature comparison that although the roasted cell coffee samples had several odor-active compounds in common with the conventionally prepared coffees, the complete aroma and flavor profile of cell coffee samples require further efforts to closely resemble conventional coffee. Above, the presence and identification of odor-active compounds have been discussed. However, the mere presence of the compounds is not enough to produce a complete replicate of conventional coffee, as the specific concentration ratios of the about 20–30 key volatiles are crucial for the overall flavor of coffee.^13^ The reported odor-active compounds and their concentrations in conventional coffee vary a lot depending on parameters such as the growth area, coffee variety, roasting method, and coffee extraction method,^11,14−17,24−26^ but there are some recurring relationships between compound groups. The contents as well as odor impacts of, for example, 4-vinylguiacol and furans such as 4-hydroxy-2,5-dimethyl-3-furanone, are typically relatively high, followed by 1–2 magnitude lower contents of guaiacol, 2-furfurylthiol and 3-methylbutanal, and finally various pyrazines each in trace amounts. In the present study, the relative contents of, for example, 4-vinylguaiacol to 3-methylbutanal and the individual pyrazines in cell coffees, followed this pattern (Supporting Information Table S6). In contrast, the lack of guaiacol in the cell coffees as discussed above and lower relative contents of 4-hydroxy-2,5-dimethyl-3-furanone in cell coffees compared to conventional coffee differentiated the samples.

Especially, some of the key odorants are lacking in cell coffee samples compared to the conventional coffee, which can be explained by several different factors. Many of the key odor-active compounds in conventional coffee are related to the Maillard reactions that occur during roasting, and therefore, the presence of precursor compounds, proteins and reducing carbohydrates, affects the formation of the odor-active compounds.^16^ Green coffee beans are composed of carbohydrates (60% Dw), lipids (10–16% Dw), proteins (10%), and chlorogenic acids (7–10% Dw) as reviewed by Moreira et al.,^50^ while plant cell cultures have been reported to contain approximately 21–37% of dietary fiber, 18–34% of free sugars, and 14–19% of protein.^32^ The chemical composition of plant cells indicates that the Maillard reaction can occur during roasting of the coffee cells. However, as reviewed by Seninde and Chambers,^11^ the formation of pyrazines and pyridines depends on the other coffee production steps such as growth and fermentation in addition to the roasting. Indeed, a similar difference could be seen when comparing the volatile profiles of the cell coffee samples to those of roasted green beans (Supporting Information Figure S5). Conventional coffee and roasted green beans had higher contents of different pyrazines than cell coffees, but especially, the conventional coffee sample was characterized by higher contents for guaiacol and maltol.

The current study focused only on a simplified proof of concept of cell-based coffee and did not include all possible processing steps (i.e., fermentation) or their optimization (e.g., roasting), meaning that a great potential remains in developing the coffee cell production and processing protocol further in order to improve the cell-based coffee.

In conclusion, the current study confirms that cell culture-derived coffee exhibits an aroma profile with similar odor-active compounds as conventional coffee even under nonoptimized process conditions. However, the absence of several key odor-active compounds of coffee indicates that further optimization is required to obtain the aroma profile characteristic to coffee. It must be noted that cell cultured coffee is regarded as novel food and requires regulatory approval in the EU and USA for commercial applications.^8^ Future studies should therefore concentrate both on toxicological and analytical examinations but also on technical aspects of coffee processing such as roasting and formulation.

## Supporting Information Available

Cell culture media (Table S1); calibration parameters for hydroxycinnamic acids/esters (Table S2); sensory attributes (Table S3); color values (Table S4); GC-MS identifications and relative peak areas of odor-active compounds (Table S5); semiquantification of volatile compounds (Table S6); distribution of the solid filter cake color values in the principal component analysis scores and loadings plots (Figure S1); distribution of the coffee beverage color values in the principal component analysis scores and loadings plots (Figure S2); spider plot of the sensory profiles (Figure S3); cumulative olfactograms (Figure S4); principal component analysis scores and loading plots of the volatile compound profiles (Figure S5) (PDF)
