# Supplementary material for: Proof of Concept for Cell Culture-Based Coffee
Source: J Agric Food Chem. 2023 Nov 16;71(47):18478–88. doi: 10.1021/acs.jafc.3c04503 (PMC10690795; doi:10.1021/acs.jafc.3c04503)
Supplement: Supplementary file 1 — jf3c04503_si_001.pdf [file jf3c04503_si_001.pdf]

# Proof of concept for cell culture-based coffee

Heikki Aisala, Elviira Kärkkäinen, Iina Jokinen, Tuulikki Seppänen-Laakso, Heiko Rischer*

VTT Technical Research Centre of Finland Ltd, P.O. Box 1000, FI-02044, Finland

## Supporting Information

**Table S1. Cell culture media.**

| **Composition** | **Concentration [mg L$^{-1}$]** | | |
|---|---|---|---|
| | Callus induction[3] medium | Callus establishment medium | Callus maintenance medium |
| Sucrose | 30000 | 30000 | 30000 |
| Phytagel | 2500 | - | |
| Gelrite | - | 3000 | 3000 |
| MS salts (M0221, Duchefa Biochemie bv)[1] | 2200 | 4300 | |
| McCown Woody Plant salts (M0219, Duchefa Biochemie bv)[2] | | | 2400 |
| Malt | 400 | - | |
| Casein | 100 | - | |
| Myoinositol | 100 | 100 | |
| $KH_2PO_4$ | - | 200 | |
| Thiamine | 10 | 1 | |
| Nicotinic acid | 1 | - | |
| Pyridoxine | 1 | - | |
| Glycine | 1 | - | |
| 2,4 D | 4.4 | 0.2 | |
| 2iP | 2 | - | |
| IBA | 1 | - | |
| NAA | | | 3 |
| BAP | | | 0.125 |
| pH | 5.8 | 5.8 | 5.8 |

[1]Murashige T. and Skoog F. A Revised Medium for Rapid Growth and Bio Assays with Tobacco Tissue Cultures. Physiol. Plant **1962** *15*, 473–497.

[2]Lloyd G. and McCown B. H. Commercially-feasible Micropropagation of Mountain Laurel, *Kalmia latifolia*, by Use of Shoot-tip Culture. Int. Plant Prop. Soc. Proc. **1980**, *30*, 421–427.

[3]Teixeira, J. B.; Junqueira, C. S.; Pereira, A. J. P. C.; Mello, R. I. S.; Silva, A. P. D.; Mundim, D. A. Multiplicação Clonal de Café (*Coffea arábica* L.) via Embriogênese Somática. *Embrapa Recursos Genéticos e Biotecnologia* **2004**, *121*, 39p.

**Table S2. Calibration parameters for hydroxycinnamic acids/esters.**

| | Hydroxycinnamic acids | Esters |
|---|---|---|
| Quantification | *Trans*-Ferulic acid was used as an external standard for calibration of phenolic acids. UV detection at 324 nm was used at a concentration range of 0.2 to 50 µg/mL. For caffeic and *p*-coumaric acids, detection at 326 and 309 nm were used, respectively. Identification was confirmed by mass detection at ESI positive and negative ion modes. | Caffeoyl-, feruloyl and *p*-coumaroyl quinic acids were quantified by using chlorogenic acid as external standard at a concentration range of 0.2 - 100 µg/mL. Mass detection in ESI negative ion mode at m/z 353, 367, 337 and 515 were used for CQAs, FQAs, *p*-CoQAs and di-CQAs. The different groups consisted of 5 CQAs, 5 FQAs, 1 *p*-CoQA and 4 di-CQAs.<br>For identification, the fragmentation and chromatographic data shown in Wu et al. 2021 (Doi: 10.1002/fsn3,2849) and Schütz et al. 2004 (Doi: 10.1021/jf049625x) were utilized. |
| Calibration curve | 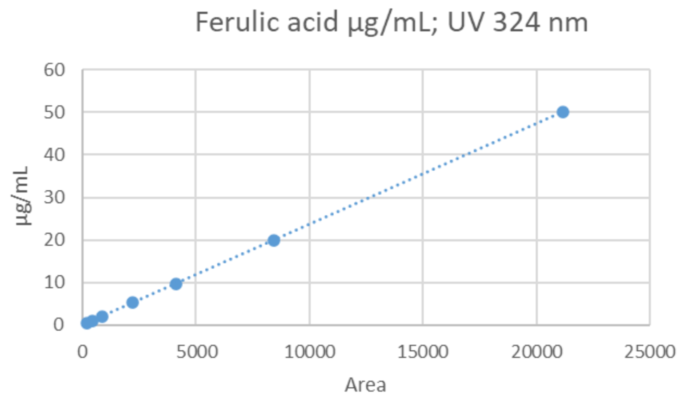  | 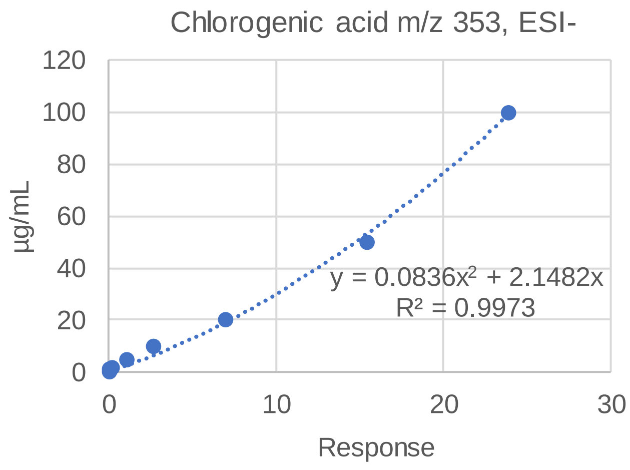  |
| Waters premier UPLC-DAD-QTof-MS conditions | Column: ACQUITY UPLC® BEH C18 1.7µm,<br>Column Temperature: 40.0 C<br>Sample Temperature: 10.0 C<br>Solvent A: 0.1%FormicA in H2O<br>Solvent B: Acetonitrile+0.1%FA<br>Run Time: 24.00 min | Column: ACQUITY UPLC® BEH C18 1.7µm,<br>Column Temperature: 40.0 C<br>Sample Temperature: 10.0 C<br>Solvent A: 1% FA in 5% MeOH<br>Solvent B: 1% FA in MeOH<br>Run Time: 24.00 min |

| | | |
|---|---|---|
| | Injection Volume (µl) - 2.00<br><br>Gradient Table<br>Time(min) Flow Rate %A %B<br>1. Initial 0.400 95.0 5.0<br>2. 18.00 0.400 70.0 30.0<br>3. 22.00 0.400 0.0 100.0<br>4. 22.10 0.400 95.0 5.0<br>5. 24.00 0.400 95.0 5.0<br><br>Waters996 PDA<br>Start Wavelength (nm) 200.00<br>End Wavelength (nm) 600.00<br>Resolution (nm) 1.2<br>Sampling Rate (spectra/s) 20.000<br><br>The MS conditions as shown for CQA etc. were used to confirm identification of phenolic acids. In addition, ESI positive ion mode runs were performed by using reserpine as Lock Spray reagent (609.2812). | Injection Volume (µl) - 2.00<br><br>Gradient Table<br>Time(min) Flow Rate %A %B<br>1. Initial 0.400 95.0 5.0<br>2. 18.00 0.400 70.0 30.0<br>3. 22.00 0.400 0.0 100.0<br>4. 22.10 0.400 95.0 5.0<br>5. 24.00 0.400 95.0 5.0<br><br>MS conditions<br>Polarity ES-<br>Analyser V Mode<br>Capillary (kV)<br>3.2<br>Sampling Cone 40.0<br>Source Temperature (°C) 150<br>Desolvation Temperature (°C) 500<br>Desolvation Gas Flow (L/Hr) 1000.0<br>Collision Energy 10.0<br>Detector 1900<br>Scan Time (sec) 0.200<br>Interscan Time (sec) 0.100<br>Start Mass 100.0<br>End Mass 2000.0<br>Data Format Centroid<br>Dynamic Range Extended<br>Lock Mass (Leucine Enkephaline) 554.2615<br><br>Waters996 PDA<br>Start Wavelength (nm) 200.00<br>End Wavelength (nm) 600.00<br>Resolution (nm) 1.2<br>Sampling Rate (spectra/s) 20.000 |

**Table S3. Sensory attributes, their definitions, used reference products and their anchored intensities on the 0-10 line scale used in the sensory profiling of cell coffee.**

| # | Attribute | Attribute description | Reference | Intensity |
|---|---|---|---|---|
| **Odour** | | | | |
| 1 | Roasted odour | Odour typical to brewed coffee | Roasted Arabica coffee beans, (Pelican rouge), 10 g | 8 |
| 2 | Burnt sugar odour/flavour | Odour typical to strongly caramelized sugar that can also be reminiscent of molasses or dark syrup | Caramelized sugar, 10 g portion: 450 g sucrose boiled for 13 min, cooled, ground coarsely, and reheated for 10 min at 50 °C before the evaluation | 6 |
| 3 | Smoky odour | - | Smoke salt, 1 g | 9 |
| 4 | Burning rubber odour | - | | |
| 5 | Tea-like odour | Odour typical to black teas such as Assam or English breakfast teas | English breakfast tea (2 pouches in 440 ml water for 3 minutes) | 8 |
| 6 | Honey odour | | Undiluted mixed flower honey, 6 g | 10 |
| 7 | Fruity odour | Fruity or berry-like odour, type specified with an open comment | | |
| **Taste and flavour** | | | | |
| 8 | Sourness | | Lightly roasted Arabica coffee (Juhla Mokka, Paulig) diluted by 20% | 9 |
| 9 | Bitterness | | Caffeine, 0.1% solution | 7 |
| 10 | Burnt sugar flavour | As above | Caramelized sugar as above | 10 |
| 6 | Honey flavour | Sweet and flowery flavour | Honey solution 4% | 6 |
| 7 | Green flavour | Flavour similar to fresh grass or dark green leafy vegetables | Minced frozen spinach melted and mixed with water 50:50, 15-20 g | 6 |

**Table S4. Color values (mean values and standard deviations) for ground green beans roasted for different durations at 225 °C.**

| Roasting time (min) | L* | | a* | | b* | |
|---|---|---|---|---|---|---|
| 6 | 36.24 | (0.04) | 5.02 | (0.11) | 4.45 | (0.08) |
| 8 | 35.60 | (0.04) | 4.37 | (0.08) | 3.75 | (0.07) |
| 10 | 34.58 | (0.07) | 2.97 | (0.12) | 2.19 | (0.05) |

**Table S5. GC-MS identifications and relative peak areas of odour active compounds of conventional coffee and cell coffee samples. UC=unroasted cell coffee, R1= roast level 1 cell coffee, R2= roast level 2 cell coffee, R3=roast level 3 cell coffee, PR=Pelican Rouge, conventional Arabica coffee**

| No | LRI[a] VF-WAX | Compound | LRI database | Main MS ions | Relative peak area (%) | | | | | Reported in coffee literature[c] |
|---|---|---|---|---|---|---|---|---|---|---|
| | | | | | UC | R1 | R2 | R3 | Ref | |
| 1 | 987 | 2,3-butanedione | 970 | 43.0, 86.0 | nd | 0.075 | 0.057 | nd | 0.111 | 11, 15, 16, 17, 25 |
| 2 | 1076 | 2,3-pentanedione | 1054 | 39.0, 43.0, 57.0, 100.0 | nd | 0.432 | 0.250 | 0.361 | 0.268 | 15, 16, 25 |
| 3 | 1111 | Hexanal | 1084 | 41.0, 43.9, 56.0, 57.0, 72.1 | nd | 0.795 | 1.974 | 0.527 | 0.066 | 17, 25 |
| 4 | 1135 | (*E*)-2-methylbut-2-enal | 1101 | 29.0, 39.0, 41.0, 55.0, 84.1 | nd | nd | nd | nd | 0.077 | 25 |
| 5 | 1272 | (Z)-hept-4-enal | 1230 | 41.0, 55.0, 68.0, 84.0 | 0.102 | 0.460 | nd | nd | nd | |
| 6 | 1309 | 2-methylpyrazine | 1266 | 39.0, 40.0, 53.0, 67.0, 94.1 | nd | nd | nd | nd | 4.227 | 11 |
| 7 | 1332 | 1-octen-3-one | 1300 | 55.0, 70.0, 27.0, 43.0 | nd | nd | nd | nd | nd | 16 |
| 8 | 1331 | 3-hydroxybutan-2-one | 1284 | 27.0, 43.0, 45.0, 88.1 | nd | nd | nd | nd | 0.019 | |
| 9 | 1364 | 2,5-dimethylpyrazine | 1320 | 39.0, 40.0, 42.0, 81.0, 108.1 | nd | nd | nd | nd | 1.857 | |
| 10 | 1365 | (*E*)-hept-2-enal | 1323 | 41.0, 55.0, 69.0, 83.0 | 0.955 | 0.460 | 1.186 | 0.353 | nd | |
| 12 | 1424 | 2-ethyl-6-methylpyrazine | 1386 | 39.0, 56.0, 67.0, 94.0, 121.0 | nd | nd | nd | nd | 1.508 | 16 |
| 13 | 1433 | 2-ethyl-5-methylpyrazine | 1387 | 39.0, 56.0, 94.1, 107.1, 121.1 | nd | nd | nd | nd | 1.094 | 11 |
| 14 | 1444 | 2,3,5-trimethylpyrazine | 1402 | 39.0, 39.9, 42.0, 81.1, 122.1 | nd | nd | nd | nd | m.p.[d] | |
| 15 | 1444 | 2-ethyl-3-methylpyrazine | 1407 | 39.0, 42.0, 81.0, 122.1 | nd | nd | nd | nd | m.p. | 16 |
| 17 | 1462 | Heptan-1-ol | 1453 | 41.0, 56.0, 70.0 | nd | 0.067 | 0.085 | 0.062 | nd | |
| 18 | 1462 | 2-propylpyrazine | 1429 | 39.0, 94.1, 107.1, 122.1 | nd | nd | nd | nd | 0.081 | |
| 23 | 1505 | (*2E,4E*)-hepta-2,4-dienal | 1495 | 39.0, 41.0, 53.0, 81.0, 110.0 | 0.432 | nd | nd | nd | nd | |
| 24 | 1504 | 1-(1-methoxypropan-2-yloxy)propan-2-ol | 1478 | 31.0, 45.0, 59.0, 73.0, 103.1 | nd | 0.094 | 0.119 | 0.046 | 0.237 | |
| 25 | 1528 | 3,5-diethyl-2-methylpyrazine | 1496 | 105.0, 122.0, 135.0, 149.0, 150.0 | nd | nd | nd | nd | 0.152 | 26 |
| 27 | 1527 | Decanal | 1498 | 41.0, 57.0, 82.1, 95.1 | 0.232 | nd | nd | nd | nd | |

| | | | | | | | | | | |
|---|---|---|---|---|---|---|---|---|---|---|
| 28 | 1559 | Furan-2-ylmethyl acetate | 1531 | 43.0, 52.0, 81.0, 98.0, 140.0 | nd | 1.106 | 0.653 | 0.885 | 7.785 | 11, 16 |
| 30 | 1588 | Benzaldehyde | 1520 | 50.0, 51.0, 77.0, 105.0, 106.0 | 5.939 | 1.202 | 1.922 | 1.009 | nd | 11, 26 |
| 34 | 1704 | 2-Phenylacetaldehyde | 1640 | 65.0, 91.0, 120.0, 135.0, | 6.350 | 0.329 | 0.561 | 0.334 | 0.194 | 15 |
| 40 | 1912 | 2-methoxyphenol (guaiacol) | 1861 | 43.0, 57.0, 87.0, 101.1 | nd | nd | nd | nd | 0.671 | 11, 16 |
| 43 | 2027 | 3-hydroxy-2-methylpyran-4-one (maltol) | 1969 | 43.0, 55.0, 71.1, 97.0, 126.0 | nd | 1.532 | nd | 1.280 | 0.383 | |
| 45 | 2055 | Pentadecanal | 2041 | 41.0, 43.0, 55.0, 57.0, 82.0, 96.0 | 8.203 | nd | nd | nd | nd | |
| 46 | 2075 | 4-hydroxy-2,5-dimethylfuran-3-one (furaneol) | 2043 | 29.0, 43.0, 57.0, 85.0, 128.0 | nd | nd | nd | nd | nd | 11 |

[a] Linear retention index [b] not detected [c] reference numbering corresponds to the order of the bibliography of the main manuscript [d] Merged peak, abundance cannot be defined

**Table S6. Semi-quantification of volatile compounds of roasted green beans, roasted conventional coffee and cell coffee samples. The amounts are expressed as 3-octanol equivalents in the sample headspace (ng/kg coffee extract).**

| LRI[a] (BPX5) | Compound | Green beans, Roast 1 | Green beans, Roast 2 | Green beans, Roast 3 | Coffee cells, Unroasted | Coffee cells, Roast 1 | Coffee cells, Roast 2 | Coffee cells, Roast 3 | Reference Arabica coffee |
|---|---|---|---|---|---|---|---|---|---|
| 599 | 2,3-Butanedione | 10–100 | 10–100 | 10–100 | 10–100 | 10–100 | 10–100 | 10–100 | 10–100 |
| 664 | 3-Methylbutanal | 10–100 | 10–100 | 10–100 | 1–10 | 10–100 | 10–100 | 10–100 | 10–100 |
| 674 | 2-Methylbutanal | 10–100 | 10–100 | 10–100 | 1–10 | 1–10 | 1–10 | 1–10 | 1–10 |
| 678 | Hydroxyacetone | 1–10 | 1–10 | 1–10 | 1–10 | <1 | <1 | <1 | 1–10 |
| 702 | 2,3-Pentanedione | 10–100 | 10–100 | 10–100 | 1–10 | 1–10 | 1–10 | 1–10 | 1–10 |
| 723 | 3-Hydroxybutanone | 10–100 | 10–100 | 10–100 | nd | 1–10 | 1–10 | 1–10 | 10–100 |
| 763 | Pyridine | 10–100 | 10–100 | 10–100 | <1 | 1–10 | 1–10 | 1–10 | 10–100 |
| 811 | Hexanal | <1 | <1 | <1 | <1 | <1 | <1 | <1 | <1 |
| 824 | Dihydro-2-methyl-3-furanone | 10–100 | 10–100 | 10–100 | nd | 1–10 | <1 | 1–10 | 100–1000 |
| 845 | 2-Methylpyrazine | 1–10 | 1–10 | 1–10 | 10–100 | 10–100 | 10–100 | 10–100 | 1–10 |
| 854 | Furfural | 1–10 | 1–10 | 1–10 | nd | 10–100 | 1–10 | 1–10 | 10–100 |
| 866 | 2-Furanmethanol | 10–100 | 10–100 | 10–100 | <1 | 1–10 | 1–10 | 1–10 | 100–1000 |
| 932 | 2-Acetylfuran | 100–1000 | 100–1000 | 100–1000 | 1–10 | 100–1000 | 100–1000 | 100–1000 | 100–1000 |
| 934 | 2,6-Dimethylpyrazine | 100–1000 | 100–1000 | 100–1000 | nd | 10–100 | 10–100 | 10–100 | 100–1000 |

| | | | | | | | | | |
|---|---|---|---|---|---|---|---|---|---|
| 938 | 2-Ethylpyrazine | 10–100 | 10–100 | 10–100 | nd | 10–100 | 10–100 | 10–100 | 100–1000 |
| 940 | 2,3-Dimethylpyrazine | 10–100 | 10–100 | 10–100 | <1 | 1–10 | 1–10 | 1–10 | 100–1000 |
| 967 | Hexanoic acid | 10–100 | 10–100 | 10–100 | nd | <1 | <1 | <1 | 10–100 |
| 986 | 5-Methylfurfural | 1–10 | 10–100 | 10–100 | nd | nd | nd | nd | 10–100 |
| 990 | Phenol | 1–10 | 1–10 | 1–10 | 100–1000 | 1–10 | 10–100 | 1–10 | 1–10 |
| 998 | Benzaldehyde | 100–1000 | 100–1000 | 100–1000 | <1 | 100–1000 | 100–1000 | 100–1000 | 100–1000 |
| 1003 | Furfuryl acetate | 1–10 | 1–10 | 1–10 | <1 | 1–10 | 1–10 | 1–10 | 10–100 |
| 1019 | 2-Ethyl-6-methylpyrazine | 10–100 | 10–100 | 10–100 | 10–100 | 10–100 | 10–100 | 10–100 | 10–100 |
| 1023 | Trimethylpyrazine | 10–100 | 10–100 | 10–100 | nd | 10–100 | 1–10 | 1–10 | 100–1000 |
| 1025 | 2-Ethyl-5-methyl-pyrazine (tentative) | 10–100 | 10–100 | 10–100 | nd | <1 | <1 | <1 | 10–100 |
| 1037 | 2-Formylpyrrole | 1–10 | 1–10 | 1–10 | nd | nd | nd | nd | 10–100 |
| 1038 | 2-Formyl-1-methylpyrrole | 10–100 | 10–100 | 10–100 | nd | <1 | <1 | <1 | 10–100 |
| 1068 | 4-Hydroxy-2,5-dimethyl-3-furanone (Furaneol) | 1–10 | 1–10 | 1–10 | nd | 1–10 | 1–10 | 1–10 | 10–100 |
| 1080 | Benzeneacetaldehyde | 1–10 | 1–10 | 1–10 | nd | 1–10 | 1–10 | 1–10 | 10–100 |
| 1096 | 2,5-Dimethyl-3-ethylpyrazine | 1–10 | <1 | 1–10 | nd | <1 | <1 | <1 | 1–10 |
| 1106 | 2-Acetyl-1-methylpyrrole | 10–100 | 10–100 | 10–100 | 10–100 | 1–10 | 10–100 | 1–10 | 10–100 |
| 1119 | 2-Methoxyphenol (Guaiacol) | 10–100 | 10–100 | 10–100 | <1 | <1 | <1 | <1 | 10–100 |
| 1149 | Maltol | 1–10 | 1–10 | 1–10 | nd | <1 | <1 | <1 | 10–100 |
| 1175 | 3,5-Diethyl-2-methylpyrazine | 1–10 | 1–10 | 1–10 | <1 | <1 | <1 | <1 | 10–100 |
| 1220 | Decanal | 1–10 | 1–10 | 1–10 | nd | 10–100 | 1–10 | 10–100 | 10–100 |
| 1255 | 5-Hydroxymethylfurfural | 1–10 | 1–10 | 1–10 | nd | nd | nd | nd | 1–10 |
| 1310 | 4-Ethyl-2-methoxyphenol | <1 | <1 | <1 | <1 | <1 | <1 | <1 | <1 |
| 1355 | 2-Methoxy-4-vinylphenol (4-Vinylguaiacol) | 10–100 | 10–100 | 10–100 | nd | 100–1000 | 100–1000 | 100–1000 | 1–10 |

[a] Linear retention index [b] not detected

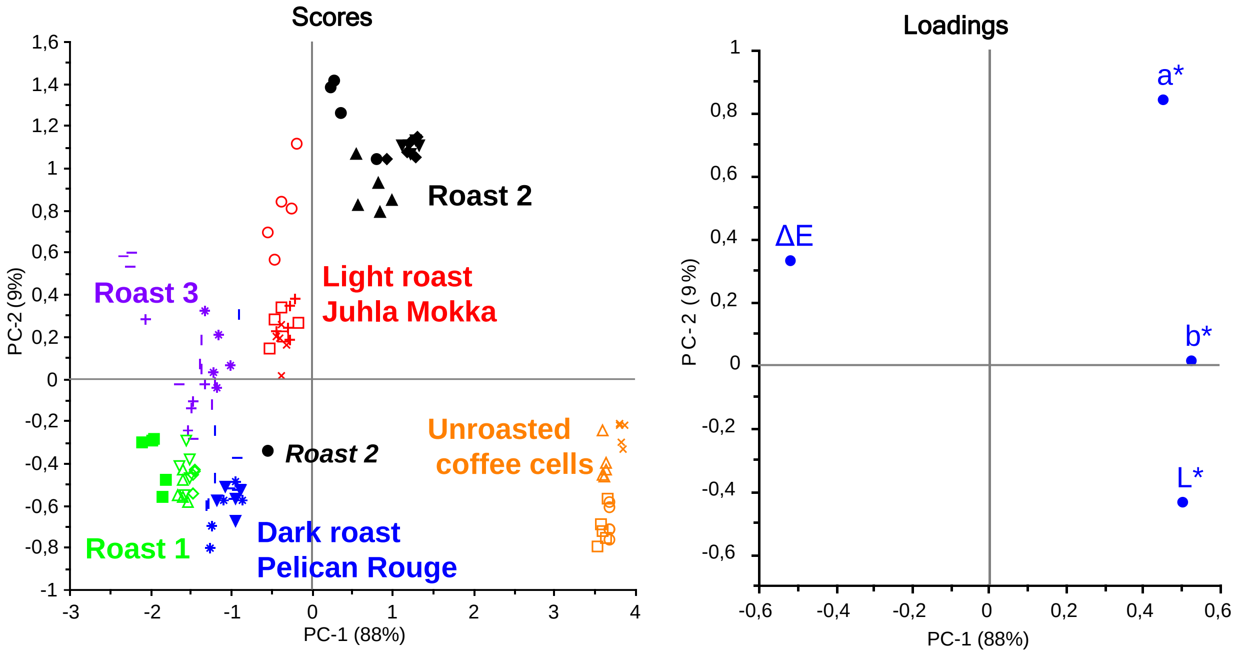


**Figure S1. Distribution of the solid filter cake color values in the Principal component analysis Scores and Loadings plots. The model is based on mean centered and standardized data. In the scores plot, each color corresponds to a different sample type, while different symbols point to different batches (total four) each with 5 measurement points.**

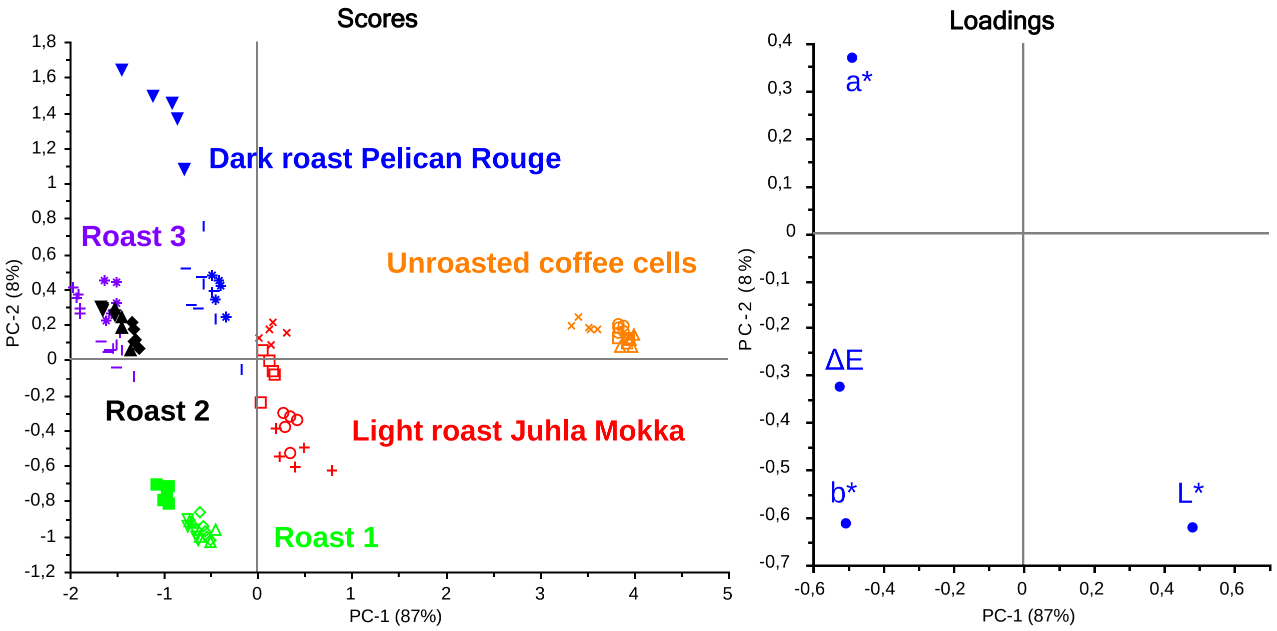


**Figure S2. Distribution of the coffee beverage color values in the Principal component analysis Scores and Loadings plots. The model is based on mean centered and standardized data. Coloring and symbols match the filter cake figure above; however, Roast 2 has only 3 batches due to a failed first brew.**

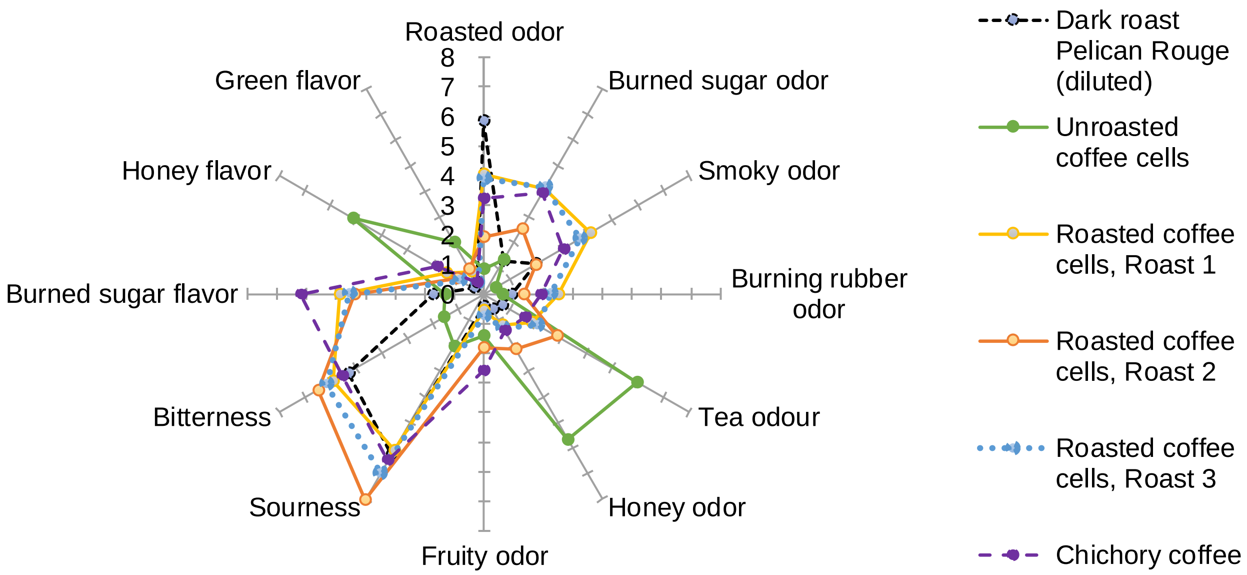


**Figure S3. Spiderplot of the sensory profiles of cell-based and conventional coffee.**

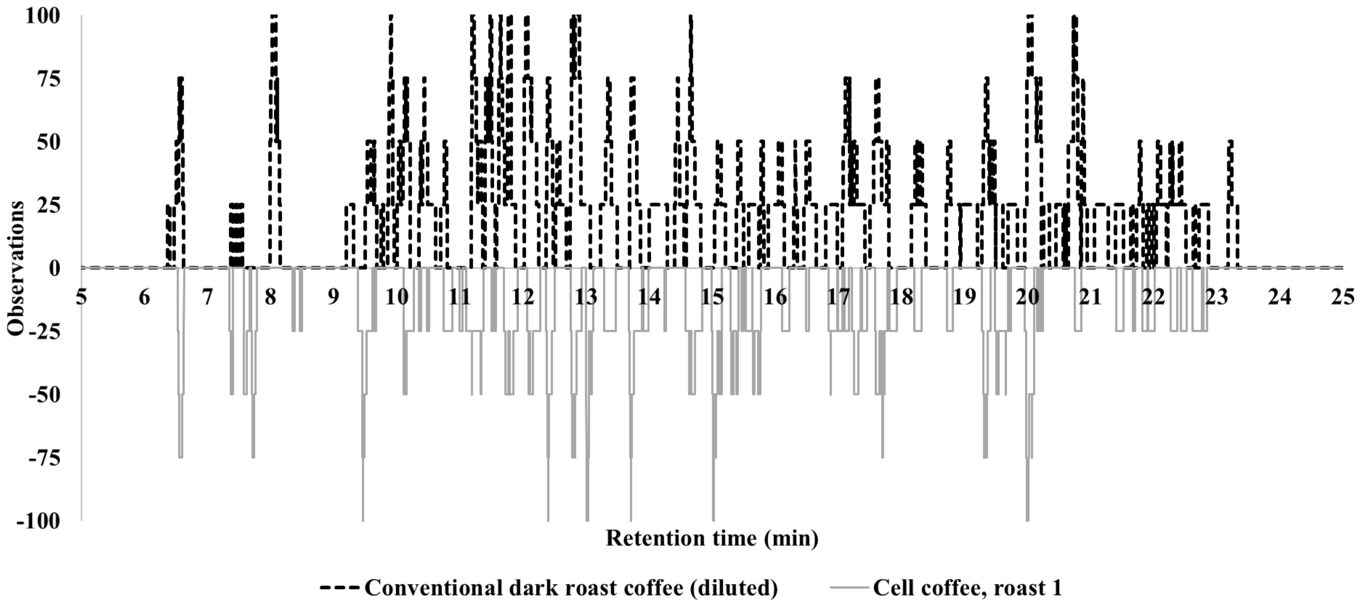


**Figure S4. Cumulative olfactograms of Dark roast Pelican Rouge (conventional Arabica coffee) and Cell coffee, Roast 1 samples.**

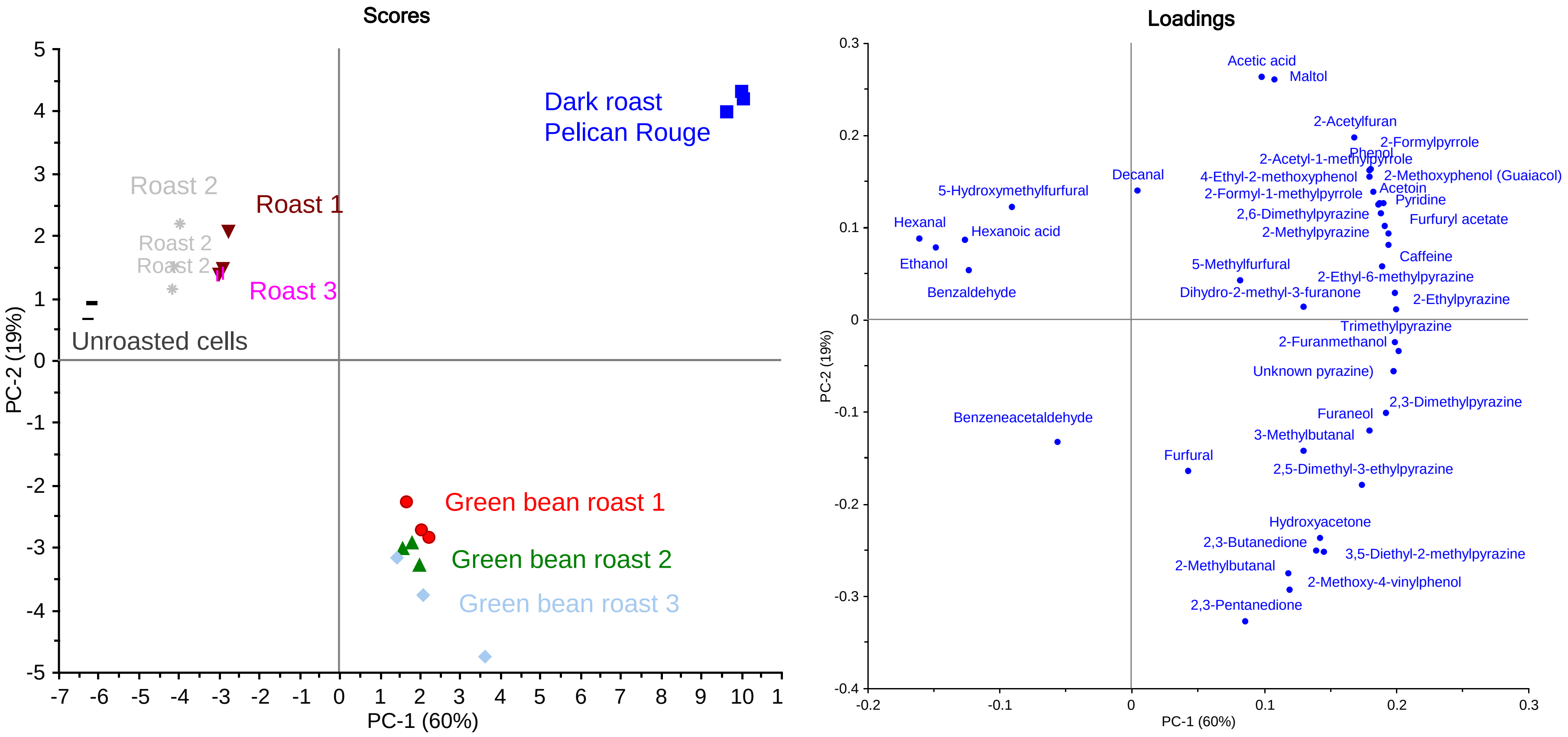


**Figure S5. Principal component analysis Scores and Loadings plots of the volatile compound profiles of different cell coffees, conventional dark roast coffee, and roasted ground green beans. The model is built with data based on volatile compound contents calculated as 3-octanol equivalents in the sample headspace measured on a BPX5 column.**
